# 4-Nitro­phenyl α-l-rhamnopyran­oside hemihydrate^1^

**DOI:** 10.1107/S1600536808006387

**Published:** 2008-03-14

**Authors:** Jianbo Zhang, Jie Fu, Xuan Chen, Yijun Gu, Jie Tang

**Affiliations:** a Department of Chemistry, East China Normal University, Shanghai 200062, People’s Republic of China; b Shanghai Innovative Research Center of Traditional Chinese Medicine, Cailun Road 720, No. 3 Building, Shanghai 201203, People’s Republic of China

## Abstract

The absolute configuration of the title compound, C_12_H_15_NO_7_·0.5H_2_O, was assigned from the synthesis. There are two rhamnoside mol­ecules and one water mol­ecule in the asymmetric unit, displaying O—H⋯O hydrogen bonding. One of the nitro groups does not conjugate efficiently with the benzene ring.

## Related literature

For related literature, see: Garegg & Norberg (1983 ▶); Garegg *et al.* (1978 ▶); Martearena *et al.* (2003 ▶); Nishio *et al.* (2004 ▶); Temeriusz *et al.* (2005 ▶); Flack (1983 ▶); Flack & Bernardinelli (2000 ▶).
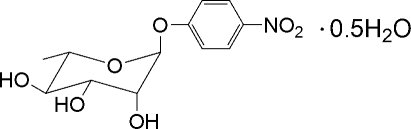


## Experimental

### 

#### Crystal data

C_12_H_15_NO_7_·0.5H_2_O
*M* *_r_* = 294.26
Monoclinic, 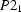

*a* = 10.6189 (10) Å
*b* = 6.9002 (7) Å
*c* = 18.9318 (18) Å
β = 100.909 (2)°
*V* = 1362.1 (2) Å^3^
*Z* = 4
Mo *K*α radiation
μ = 0.12 mm^−1^
*T* = 293 (2) K
0.51 × 0.49 × 0.31 mm

#### Data collection

Bruker SMART CCD area-detector diffractometer
Absorption correction: multi-scan (*SADABS*; Sheldrick, 1996 ▶) *T*_min_ = 0.802, *T*_max_ = 1.000 (expected range = 0.772–0.963)
8073 measured reflections
3220 independent reflections
2745 reflections with *I* > 2σ(*I*)
*R*_int_ = 0.087

#### Refinement

*R*[*F* ^2^ > 2σ(*F* ^2^)] = 0.042
*wR*(*F* ^2^) = 0.092
*S* = 0.97
3220 reflections
405 parameters
12 restraints
H atoms treated by a mixture of independent and constrained refinement
Δρ_max_ = 0.20 e Å^−3^
Δρ_min_ = −0.21 e Å^−3^

### 

Data collection: *SMART* (Bruker, 2003 ▶); cell refinement: *SMART*; data reduction: *SAINT* (Sheldrick, 2008 ▶) and *SHELXTL* (Sheldrick, 2008 ▶); program(s) used to solve structure: *SHELXS97* (Sheldrick, 2008 ▶); program(s) used to refine structure: *SHELXL97* (Sheldrick, 2008 ▶); molecular graphics: *SHELXTL*; software used to prepare material for publication: *SHELXTL*.

## Supplementary Material

Crystal structure: contains datablocks I, global. DOI: 10.1107/S1600536808006387/av2007sup1.cif

Structure factors: contains datablocks I. DOI: 10.1107/S1600536808006387/av2007Isup2.hkl

Additional supplementary materials:  crystallographic information; 3D view; checkCIF report

## Figures and Tables

**Table 1:** Hydrogen-bond geometry (Å, °)

*D*—H⋯*A*	*D*—H	H⋯*A*	*D*⋯*A*	*D*—H⋯*A*
O2—H2*A*⋯O15^i^	0.87 (4)	1.83 (4)	2.697 (3)	171 (4)
O3—H3*A*⋯O4^ii^	0.87 (4)	1.78 (4)	2.652 (3)	179 (3)
O4—H4*A*⋯O10	0.829 (19)	1.98 (2)	2.799 (3)	168 (3)
O9—H9*A*⋯O11^iii^	0.80 (4)	1.96 (4)	2.724 (3)	161 (3)
O10—H10⋯O3^ii^	0.828 (19)	2.18 (2)	2.993 (3)	166 (3)
O11—H11*A*⋯O3^ii^	0.816 (19)	1.92 (2)	2.668 (3)	153 (3)
O15—H15*A*⋯O9	0.89 (2)	2.04 (2)	2.909 (3)	165 (5)
O15—H15*B*⋯O2^ii^	0.88 (2)	1.96 (2)	2.820 (3)	167 (5)

Symmetry codes: (i) 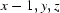; (ii) 
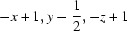
; (iii) 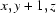.

**Fig. 1.**
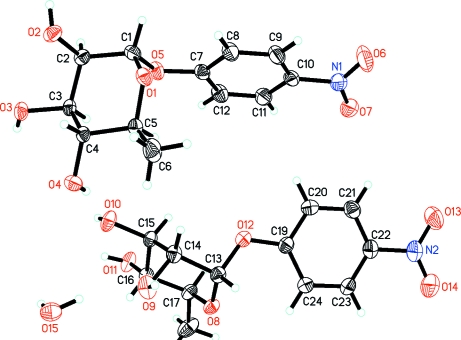
The molecular structure of (3), with displacement ellipsoids drawn at the 50% probability level. H-atom radii are arbitrary.

**Fig. 2.**
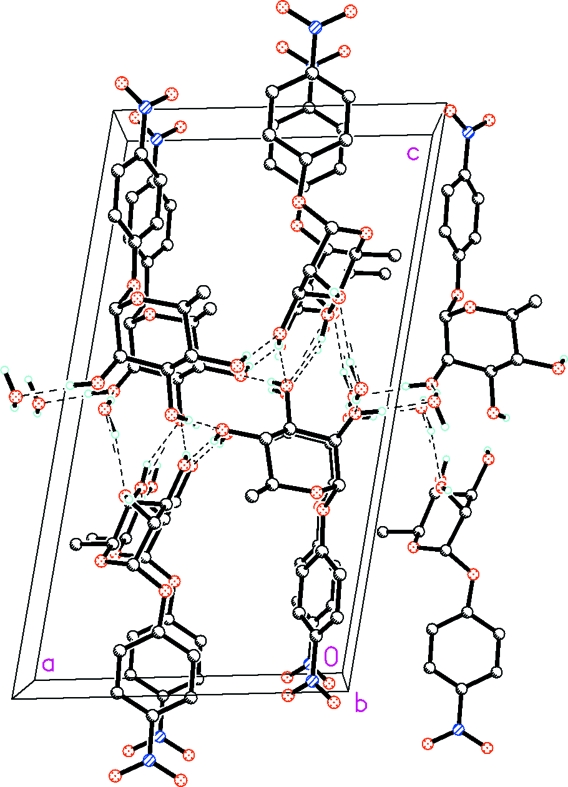
Packing diagram of (3) viewed down the b axis. Hydrogen bonds are displayed with dashed lines.

**Fig. 3.**
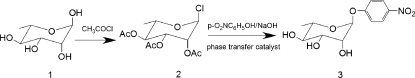
Scheme 1. The two-step synthesis of (3), with phase transfer catalysis.

## supplementary crystallographic information

### Comment

Para-nitrophenyl-α-*L*-rhamnoside is an important substrate in the studies on α-*L*-rhamnosidase, for its chromogenic property of the released *para*-nitrophenol (Garegg *et al.*, 1978). It also serves as synthetic intermediate for glycosidic compounds (Martearena *et al.*, 2003).

In order to develop a greener synthetic method, a series of approaches have been carried out in this lab. A fairly convenient route was found finally, in which the title compound was synthesized in only two steps. First, *L*-rhamnose (1) was acetylated and chlorinated to yield 2,3,4-tri-*O*-acetyl-α-*L*-rhamnopyranosyl chloride (2) in the presence of acetyl chloride; then it was converted to the target molecule (3) in the condition of phase transfer catalyst (Scheme 1). The synthetic route was more concise compared with published methods (Garegg & Norberg, 1983). Additionally, the bioactivity of the synthetic compound was confirmed by enzymatic assay (Nishio *et al.*, 2004)

Suitable crystals of target product were obtained by slow crystallization from 95% ethanol. The crystal structure was determined in order to ascertain its stereochemistry and solid-state conformation. These data are consistent with the proton and carbon NMR studies. Due to the absence of heavy atoms, refinement of the Flack parameter was not possible, and the absolute configurations could not be determined directly. Instead, they were assigned based on the knowledge of stereochemistry of the synthetic precursors and the mechanisms of synthesis. The crystal of rhamnoside has two molecules and one water molecule in the independent part of the unit cell. The configuration, conformation and atom numbering are shown in Fig. 1.

Similar to the known structures of the nitrophenyl glycopyranosides, the analyzed rhamnopyranoside (3) crystallizes in the *P *2_1_ space group. Besides, one of the nitro groups is slightly rotated with respect to the phenyl fragments. The angles between the best planes of the phenyl ring and the nitro groups are 13.3° and 0.5°, respectively. This finding partly supports the earlier opinion that the nitro group does not conjugate effectively with the benzene ring (Temeriusz *et al.*, 2005). The sugar moieties adopt ^4^C_1_ conformations. Fig. 2 shows the intermolecular interactions in the crystal lattice. The crystal structure of (3) consists of molecular sheets lying perpendicular to the *b* axis (Fig. 2), in which the molecules are linked by short hydrogen bonds (Table 1).

For related literature, see [type here to add references to related literature].

### Experimental

Para-nitrophenyl-α-*L*-rhamnoside (3) was obtained upon one-pot reaction combined with glycosylation and deacetylation, using 10%NaOH aqueous and cetyl alkyl trimethyl ammonium bromide from 2,3,4-tri-*O*-acetyl-α-*L*-rhamnosyl chloride and *para*-nitrophenol. A yield of 37% of the title compound was obtained after purification by flash column chromatography on silica gel with petroleum ether–ethyl acetate (1:3) as solvent. The compound was then recrystallized *via* solvent evaporation (ethanol) at room temperature, appearing as colorless blocks. Analysis: Mp: 179–180°C, [α]^D^_20_ -158.7° (c 1.0, EtOH) Rf 0.49 (dichloromethane/ methanol, 8:1, silica-gel plate 60 F_254_); 1*H*-NMR (CD_3_OD, 500 MHz, p.p.m.): δ 8.22(2*H*, aromatic H), 7.25(2*H*, aromatic H), 5.60(d, 1H, J1, 2=2 Hz, H-1), 4.03(m, 1H, H-2), 3.84(dd, 1H, H-3), 3.56–3.36(m, 2H, H-4, H-5), 1.22(d, 3H, CH3); 13 C-NMR (125 MHz, CD3OD): δ 150.83, 141.85, 124.75, 115.62(aromatic C), 98.01(C-1), 71.62, 70.15, 69.75, 69.34(C-2, C-3, C-4, C-5), 16.07(C-6).

### Refinement

In the absence of any significant anomalous scattering, the Flack (1983) parameter was indeterminable (Flack & Bernardinelli, 2000). Hence, the Friedel equivalents were merged prior to the final refinements, and the absolute structure was set by reference to the known chirality of the enantiopure starting sugar employed.

### Crystal data

**Table:**

C_12_H_15_NO_7_·0.5H_2_O	*F*_000_ = 620
*M**_r_* = 294.26	*D*_x_ = 1.435 Mg m^−^^3^
Monoclinic, *P*2_1_	Melting point: 453 K
Hall symbol: P 2yb	Mo *K*α radiation λ = 0.71073 Å
*a* = 10.6189 (10) Å	Cell parameters from 3190 reflections
*b* = 6.9002 (7) Å	θ = 4.8–5.7º
*c* = 18.9318 (18) Å	µ = 0.12 mm^−^^1^
β = 100.909 (2)º	*T* = 293 (2) K
*V* = 1362.1 (2) Å^3^	Prismatic, colourless
*Z* = 4	0.51 × 0.49 × 0.31 mm

### Data collection

**Table:**

Bruker SMART CCD area-detector diffractometer	3220 independent reflections
Radiation source: fine-focus sealed tube	2745 reflections with *I* > 2σ(*I*)
Monochromator: graphite	*R*_int_ = 0.087
*T* = 293(2) K	θ_max_ = 27.0º
φ and ω scans	θ_min_ = 2.0º
Absorption correction: multi-scan(SADABS; Sheldrick, 1996)	*h* = −12→13
*T*_min_ = 0.802, *T*_max_ = 1.000	*k* = −8→7
8073 measured reflections	*l* = −23→24

### Refinement

**Table:**

Refinement on *F*^2^	Hydrogen site location: inferred from neighbouring sites
Least-squares matrix: full	H atoms treated by a mixture of independent and constrained refinement
*R*[*F*^2^ > 2σ(*F*^2^)] = 0.042	*w* = 1/[σ^2^(*F*_o_^2^) + (0.037*P*)^2^] where *P* = (*F*_o_^2^ + 2*F*_c_^2^)/3
*wR*(*F*^2^) = 0.092	(Δ/σ)_max_ = 0.004
*S* = 0.97	Δρ_max_ = 0.20 e Å^−^^3^
3220 reflections	Δρ_min_ = −0.21 e Å^−^^3^
405 parameters	Extinction correction: SHELXL97 (Sheldrick, 2008), Fc^*^=kFc[1+0.001xFc^2^λ^3^/sin(2θ)]^-1/4^
12 restraints	Extinction coefficient: 0.0202 (19)
Primary atom site location: structure-invariant direct methods	Absolute structure: Flack (1983)
Secondary atom site location: difference Fourier map	

### Special details

**Table:**

Experimental. Para-nitrophenyl-α-*L*-rhamnoside(3) was obtained upon one-pot reaction combined with glycosylation and deacetylation, using 10% NaOH aqueous and cetyl alkyl trimethyl ammonium bromide from 2,3,4-tri-*O*-acetyl-α-*L*-rhamnosyl chloride and *para*-nitrophenol. A yield of 37% of the title compound was obtained after purification by flash column chromatography on silica gel with Petroleum ether – Ethyl acetate (1:3) as solvent. The compound was then recrystallized *via* solvent evaporation (ethanol) at room temperature, appearing as colorless blocks. Analysis: Rf 0.49 (Dichloromethane/ methanol, 8:1, silica-gel plate 60 F254); 1*H*-NMR (CD3OD, 500 MHz, p.p.m.): δ 8.22(2*H*, aromatic H), 7.25(2*H*, aromatic H), 5.60(d, 1H, J1, 2=2 Hz, H-1), 4.03(m, 1H, H-2), 3.84(dd, 1H, H-3), 3.56–3.36(m, 2H, H-4, H-5), 1.22(d, 3H, CH3); 13 C-NMR (125 MHz, CD3OD): δ 150.83, 141.85, 124.75, 115.62(aromatic C), 98.01(C-1), 71.62, 70.15, 69.75, 69.34(C-2, C-3, C-4, C-5), 16.07(C-6).
Geometry. All e.s.d.'s (except the e.s.d. in the dihedral angle between two l.s. planes) are estimated using the full covariance matrix. The cell e.s.d.'s are taken into account individually in the estimation of e.s.d.'s in distances, angles and torsion angles; correlations between e.s.d.'s in cell parameters are only used when they are defined by crystal symmetry. An approximate (isotropic) treatment of cell e.s.d.'s is used for estimating e.s.d.'s involving l.s. planes.
Refinement. Refinement of *F*^2^ against ALL reflections. The weighted *R*-factor *wR* and goodness of fit *S* are based on *F*^2^, conventional *R*-factors *R* are based on *F*, with *F* set to zero for negative *F*^2^. The threshold expression of *F*^2^ > σ(*F*^2^) is used only for calculating *R*-factors(gt) *etc*. and is not relevant to the choice of reflections for refinement. *R*-factors based on *F*^2^ are statistically about twice as large as those based on *F*, and *R*– factors based on ALL data will be even larger.

### Fractional atomic coordinates and isotropic or equivalent isotropic displacement parameters (Å^2^)

**Table:**

	*x*	*y*	*z*	*U*_iso_*/*U*_eq_	
O1	0.19455 (17)	1.0324 (2)	0.33890 (9)	0.0398 (4)	
O2	0.1174 (2)	0.9293 (3)	0.47272 (10)	0.0457 (5)	
O3	0.33922 (18)	0.7408 (3)	0.52541 (9)	0.0415 (4)	
O4	0.51029 (17)	0.9318 (3)	0.44976 (10)	0.0411 (4)	
O5	0.16218 (18)	0.7063 (2)	0.30768 (9)	0.0425 (4)	
O6	0.0339 (3)	0.8596 (5)	−0.02333 (13)	0.0908 (9)	
O7	0.1729 (2)	0.6342 (4)	−0.01726 (11)	0.0666 (7)	
O8	0.82087 (17)	0.6288 (2)	0.22589 (9)	0.0403 (4)	
O9	0.7702 (2)	0.8782 (3)	0.33951 (12)	0.0528 (6)	
O10	0.6155 (2)	0.6111 (3)	0.39139 (10)	0.0482 (5)	
O11	0.7502 (2)	0.2697 (2)	0.35244 (11)	0.0468 (5)	
O12	0.60585 (18)	0.6789 (3)	0.17194 (10)	0.0471 (5)	
O13	0.4597 (3)	0.6372 (5)	−0.16104 (13)	0.0842 (8)	
O14	0.6635 (3)	0.6568 (6)	−0.14965 (14)	0.1026 (11)	
O15	0.8807 (2)	0.8258 (3)	0.49102 (14)	0.0577 (6)	
N1	0.1080 (2)	0.7455 (4)	0.01055 (13)	0.0540 (7)	
N2	0.5671 (3)	0.6494 (4)	−0.12425 (14)	0.0606 (7)	
C1	0.1293 (2)	0.8625 (4)	0.34999 (14)	0.0378 (6)	
H1	0.0367	0.8859	0.3373	0.045*	
C2	0.1625 (2)	0.7922 (4)	0.42766 (14)	0.0358 (5)	
H2	0.1225	0.6659	0.4321	0.043*	
C3	0.3070 (2)	0.7757 (4)	0.45007 (12)	0.0331 (5)	
H3	0.3366	0.6660	0.4247	0.040*	
C4	0.3743 (2)	0.9566 (4)	0.43259 (12)	0.0312 (5)	
H4	0.3514	1.0619	0.4625	0.037*	
C5	0.3317 (2)	1.0148 (4)	0.35410 (13)	0.0361 (6)	
H5	0.3574	0.9137	0.3233	0.043*	
C6	0.3851 (3)	1.2050 (5)	0.33591 (17)	0.0627 (9)	
H6A	0.3556	1.2323	0.2858	0.094*	
H6B	0.4771	1.1991	0.3460	0.094*	
H6C	0.3569	1.3056	0.3643	0.094*	
C7	0.1446 (2)	0.7275 (4)	0.23480 (13)	0.0380 (6)	
C8	0.0812 (3)	0.8810 (4)	0.19674 (15)	0.0483 (7)	
H8	0.0478	0.9806	0.2207	0.058*	
C9	0.0680 (3)	0.8846 (5)	0.12280 (16)	0.0511 (7)	
H9	0.0243	0.9858	0.0964	0.061*	
C10	0.1192 (3)	0.7394 (4)	0.08867 (14)	0.0443 (6)	
C11	0.1813 (3)	0.5855 (5)	0.12565 (15)	0.0496 (7)	
H11	0.2150	0.4869	0.1013	0.060*	
C12	0.1930 (3)	0.5790 (4)	0.19884 (15)	0.0487 (7)	
H12	0.2336	0.4743	0.2245	0.058*	
C13	0.7158 (2)	0.7509 (4)	0.21975 (14)	0.0393 (6)	
H13	0.7388	0.8774	0.2023	0.047*	
C14	0.6727 (3)	0.7788 (4)	0.29176 (14)	0.0408 (6)	
H14	0.5930	0.8540	0.2846	0.049*	
C15	0.6516 (2)	0.5824 (4)	0.32289 (13)	0.0358 (5)	
H15	0.5806	0.5181	0.2908	0.043*	
C16	0.7705 (3)	0.4593 (3)	0.32767 (13)	0.0354 (5)	
H16	0.8422	0.5205	0.3603	0.042*	
C17	0.8030 (3)	0.4388 (4)	0.25371 (14)	0.0396 (6)	
H17	0.7318	0.3744	0.2218	0.047*	
C18	0.9237 (3)	0.3276 (5)	0.2535 (2)	0.0662 (10)	
H18A	0.9419	0.3265	0.2057	0.099*	
H18B	0.9132	0.1970	0.2688	0.099*	
H18C	0.9933	0.3879	0.2857	0.099*	
C19	0.6052 (3)	0.6749 (4)	0.09986 (14)	0.0413 (6)	
C20	0.4846 (3)	0.6600 (4)	0.05670 (15)	0.0466 (6)	
H20	0.4122	0.6551	0.0776	0.056*	
C21	0.4716 (3)	0.6523 (4)	−0.01641 (15)	0.0491 (7)	
H21	0.3908	0.6428	−0.0455	0.059*	
C22	0.5802 (3)	0.6590 (4)	−0.04625 (15)	0.0473 (7)	
C23	0.7006 (3)	0.6735 (5)	−0.00431 (16)	0.0505 (7)	
H23	0.7725	0.6778	−0.0256	0.061*	
C24	0.7141 (3)	0.6817 (5)	0.06912 (16)	0.0497 (7)	
H24	0.7950	0.6916	0.0980	0.060*	
H10	0.617 (4)	0.501 (3)	0.4090 (18)	0.074 (12)*	
H2A	0.038 (4)	0.906 (6)	0.476 (2)	0.081 (12)*	
H3A	0.389 (3)	0.639 (5)	0.5343 (16)	0.052 (9)*	
H4A	0.530 (3)	0.830 (4)	0.4316 (18)	0.062 (10)*	
H9A	0.753 (3)	0.991 (5)	0.3344 (17)	0.053 (10)*	
H11A	0.743 (3)	0.284 (5)	0.3943 (11)	0.056 (9)*	
H15A	0.835 (5)	0.829 (8)	0.4467 (15)	0.124 (19)*	
H15B	0.873 (5)	0.708 (4)	0.507 (3)	0.108 (17)*	

### Atomic displacement parameters (Å^2^)

**Table:**

	*U*^11^	*U*^22^	*U*^33^	*U*^12^	*U*^13^	*U*^23^
O1	0.0446 (10)	0.0357 (9)	0.0357 (9)	0.0069 (8)	−0.0009 (8)	0.0013 (7)
O2	0.0424 (11)	0.0504 (11)	0.0482 (11)	−0.0037 (9)	0.0185 (9)	−0.0139 (9)
O3	0.0527 (11)	0.0429 (11)	0.0291 (9)	0.0109 (9)	0.0084 (7)	0.0060 (8)
O4	0.0327 (9)	0.0420 (11)	0.0469 (11)	0.0014 (8)	0.0034 (8)	−0.0095 (9)
O5	0.0499 (10)	0.0408 (10)	0.0334 (9)	0.0045 (8)	−0.0005 (8)	−0.0050 (8)
O6	0.106 (2)	0.120 (2)	0.0416 (13)	0.0504 (19)	0.0000 (13)	0.0104 (15)
O7	0.0579 (13)	0.0952 (18)	0.0489 (12)	0.0037 (13)	0.0159 (10)	−0.0109 (13)
O8	0.0423 (10)	0.0368 (10)	0.0447 (10)	−0.0003 (8)	0.0162 (7)	0.0064 (8)
O9	0.0800 (16)	0.0245 (10)	0.0513 (12)	0.0015 (10)	0.0053 (11)	−0.0015 (9)
O10	0.0726 (13)	0.0375 (11)	0.0408 (11)	0.0125 (10)	0.0268 (9)	0.0006 (9)
O11	0.0786 (14)	0.0260 (9)	0.0419 (11)	0.0029 (9)	0.0274 (10)	0.0003 (8)
O12	0.0444 (10)	0.0567 (12)	0.0417 (10)	−0.0047 (9)	0.0121 (8)	0.0030 (9)
O13	0.0877 (19)	0.104 (2)	0.0528 (14)	−0.0079 (17)	−0.0082 (13)	−0.0063 (14)
O14	0.099 (2)	0.143 (3)	0.0490 (14)	−0.013 (2)	0.0223 (14)	−0.0026 (18)
O15	0.0538 (13)	0.0506 (14)	0.0698 (16)	−0.0070 (10)	0.0146 (11)	0.0015 (12)
N1	0.0492 (14)	0.0716 (18)	0.0410 (13)	−0.0021 (14)	0.0081 (11)	−0.0081 (13)
N2	0.0780 (19)	0.0592 (17)	0.0447 (14)	−0.0011 (15)	0.0115 (14)	−0.0001 (13)
C1	0.0326 (13)	0.0405 (14)	0.0383 (14)	0.0026 (11)	0.0020 (10)	−0.0061 (11)
C2	0.0390 (13)	0.0328 (12)	0.0369 (13)	−0.0028 (11)	0.0106 (10)	−0.0042 (10)
C3	0.0416 (13)	0.0326 (12)	0.0255 (11)	0.0045 (10)	0.0073 (9)	−0.0017 (9)
C4	0.0357 (12)	0.0309 (11)	0.0269 (11)	0.0042 (10)	0.0058 (9)	−0.0040 (10)
C5	0.0388 (14)	0.0389 (13)	0.0300 (12)	−0.0003 (11)	0.0050 (10)	−0.0008 (10)
C6	0.072 (2)	0.062 (2)	0.0493 (18)	−0.0159 (17)	0.0014 (15)	0.0200 (15)
C7	0.0358 (12)	0.0406 (14)	0.0353 (13)	−0.0038 (11)	0.0005 (10)	−0.0071 (11)
C8	0.0514 (17)	0.0513 (16)	0.0401 (15)	0.0151 (13)	0.0032 (12)	−0.0059 (13)
C9	0.0509 (17)	0.0573 (18)	0.0412 (15)	0.0139 (14)	−0.0008 (12)	−0.0010 (13)
C10	0.0366 (13)	0.0590 (18)	0.0348 (13)	−0.0037 (13)	0.0005 (10)	−0.0077 (13)
C11	0.0507 (16)	0.0540 (17)	0.0442 (15)	0.0076 (14)	0.0091 (12)	−0.0118 (14)
C12	0.0539 (17)	0.0430 (16)	0.0459 (16)	0.0101 (13)	0.0007 (12)	−0.0029 (13)
C13	0.0421 (14)	0.0354 (13)	0.0408 (14)	0.0015 (11)	0.0089 (11)	0.0062 (11)
C14	0.0502 (15)	0.0343 (13)	0.0389 (14)	0.0081 (12)	0.0109 (11)	0.0037 (11)
C15	0.0432 (14)	0.0338 (13)	0.0323 (12)	0.0020 (11)	0.0119 (10)	−0.0033 (10)
C16	0.0475 (15)	0.0235 (11)	0.0367 (13)	0.0017 (10)	0.0119 (10)	−0.0020 (10)
C17	0.0478 (15)	0.0318 (13)	0.0437 (14)	0.0003 (12)	0.0201 (12)	−0.0017 (11)
C18	0.074 (2)	0.0503 (19)	0.086 (3)	0.0198 (17)	0.0460 (19)	0.0148 (17)
C19	0.0458 (14)	0.0382 (14)	0.0411 (14)	−0.0006 (12)	0.0115 (11)	0.0042 (12)
C20	0.0394 (14)	0.0477 (16)	0.0538 (17)	−0.0025 (12)	0.0113 (12)	−0.0008 (13)
C21	0.0503 (16)	0.0457 (16)	0.0484 (17)	−0.0041 (13)	0.0019 (13)	−0.0022 (13)
C22	0.0608 (17)	0.0393 (15)	0.0409 (15)	−0.0055 (13)	0.0072 (13)	0.0002 (12)
C23	0.0504 (16)	0.0579 (18)	0.0456 (15)	−0.0014 (14)	0.0151 (13)	0.0103 (14)
C24	0.0417 (14)	0.0628 (18)	0.0443 (15)	−0.0049 (14)	0.0075 (12)	0.0069 (14)

### Geometric parameters (Å, °)

**Table:**

O1—C1	1.398 (3)	C5—H5	0.9800
O1—C5	1.435 (3)	C6—H6A	0.9600
O2—C2	1.417 (3)	C6—H6B	0.9600
O2—H2A	0.87 (4)	C6—H6C	0.9600
O3—C3	1.423 (3)	C7—C12	1.382 (4)
O3—H3A	0.87 (4)	C7—C8	1.382 (4)
O4—C4	1.430 (3)	C8—C9	1.380 (4)
O4—H4A	0.829 (19)	C8—H8	0.9300
O5—C7	1.365 (3)	C9—C10	1.360 (4)
O5—C1	1.425 (3)	C9—H9	0.9300
O6—N1	1.208 (4)	C10—C11	1.370 (4)
O7—N1	1.216 (3)	C11—C12	1.368 (4)
O8—C13	1.385 (3)	C11—H11	0.9300
O8—C17	1.439 (3)	C12—H12	0.9300
O9—C14	1.416 (4)	C13—C14	1.530 (4)
O9—H9A	0.80 (4)	C13—H13	0.9800
O10—C15	1.434 (3)	C14—C15	1.512 (4)
O10—H10	0.828 (19)	C14—H14	0.9800
O11—C16	1.420 (3)	C15—C16	1.510 (4)
O11—H11A	0.816 (19)	C15—H15	0.9800
O12—C19	1.363 (3)	C16—C17	1.511 (4)
O12—C13	1.425 (3)	C16—H16	0.9800
O13—N2	1.221 (4)	C17—C18	1.494 (4)
O14—N2	1.211 (4)	C17—H17	0.9800
O15—H15A	0.89 (2)	C18—H18A	0.9600
O15—H15B	0.88 (2)	C18—H18B	0.9600
N1—C10	1.462 (3)	C18—H18C	0.9600
N2—C22	1.458 (4)	C19—C20	1.387 (4)
C1—C2	1.525 (4)	C19—C24	1.390 (4)
C1—H1	0.9800	C20—C21	1.366 (4)
C2—C3	1.517 (3)	C20—H20	0.9300
C2—H2	0.9800	C21—C22	1.378 (4)
C3—C4	1.506 (3)	C21—H21	0.9300
C3—H3	0.9800	C22—C23	1.375 (4)
C4—C5	1.523 (3)	C23—C24	1.371 (4)
C4—H4	0.9800	C23—H23	0.9300
C5—C6	1.495 (4)	C24—H24	0.9300
			
C1—O1—C5	114.34 (18)	C9—C10—N1	119.7 (3)
C2—O2—H2A	111 (3)	C11—C10—N1	118.6 (3)
C3—O3—H3A	111 (2)	C12—C11—C10	119.1 (3)
C4—O4—H4A	110 (2)	C12—C11—H11	120.5
C7—O5—C1	119.09 (19)	C10—C11—H11	120.5
C13—O8—C17	115.16 (19)	C11—C12—C7	120.2 (3)
C14—O9—H9A	106 (2)	C11—C12—H12	119.9
C15—O10—H10	104 (3)	C7—C12—H12	119.9
C16—O11—H11A	105 (2)	O8—C13—O12	113.1 (2)
C19—O12—C13	119.4 (2)	O8—C13—C14	111.9 (2)
H15A—O15—H15B	107 (5)	O12—C13—C14	105.2 (2)
O6—N1—O7	123.2 (3)	O8—C13—H13	108.8
O6—N1—C10	118.4 (3)	O12—C13—H13	108.8
O7—N1—C10	118.4 (3)	C14—C13—H13	108.8
O14—N2—O13	123.0 (3)	O9—C14—C15	109.3 (2)
O14—N2—C22	118.3 (3)	O9—C14—C13	108.9 (2)
O13—N2—C22	118.7 (3)	C15—C14—C13	109.0 (2)
O1—C1—O5	111.6 (2)	O9—C14—H14	109.9
O1—C1—C2	112.4 (2)	C15—C14—H14	109.9
O5—C1—C2	105.4 (2)	C13—C14—H14	109.9
O1—C1—H1	109.1	O10—C15—C16	112.8 (2)
O5—C1—H1	109.1	O10—C15—C14	108.3 (2)
C2—C1—H1	109.1	C16—C15—C14	110.1 (2)
O2—C2—C3	108.7 (2)	O10—C15—H15	108.5
O2—C2—C1	109.0 (2)	C16—C15—H15	108.5
C3—C2—C1	109.3 (2)	C14—C15—H15	108.5
O2—C2—H2	109.9	O11—C16—C15	111.1 (2)
C3—C2—H2	109.9	O11—C16—C17	107.17 (19)
C1—C2—H2	109.9	C15—C16—C17	109.4 (2)
O3—C3—C4	109.06 (19)	O11—C16—H16	109.7
O3—C3—C2	109.38 (19)	C15—C16—H16	109.7
C4—C3—C2	111.9 (2)	C17—C16—H16	109.7
O3—C3—H3	108.8	O8—C17—C18	107.1 (2)
C4—C3—H3	108.8	O8—C17—C16	108.83 (19)
C2—C3—H3	108.8	C18—C17—C16	113.4 (2)
O4—C4—C3	110.61 (19)	O8—C17—H17	109.1
O4—C4—C5	110.74 (19)	C18—C17—H17	109.1
C3—C4—C5	111.57 (19)	C16—C17—H17	109.1
O4—C4—H4	107.9	C17—C18—H18A	109.5
C3—C4—H4	107.9	C17—C18—H18B	109.5
C5—C4—H4	107.9	H18A—C18—H18B	109.5
O1—C5—C6	107.1 (2)	C17—C18—H18C	109.5
O1—C5—C4	108.70 (19)	H18A—C18—H18C	109.5
C6—C5—C4	113.6 (2)	H18B—C18—H18C	109.5
O1—C5—H5	109.1	O12—C19—C20	114.9 (2)
C6—C5—H5	109.1	O12—C19—C24	124.8 (2)
C4—C5—H5	109.1	C20—C19—C24	120.3 (3)
C5—C6—H6A	109.5	C21—C20—C19	120.3 (3)
C5—C6—H6B	109.5	C21—C20—H20	119.8
H6A—C6—H6B	109.5	C19—C20—H20	119.8
C5—C6—H6C	109.5	C20—C21—C22	118.8 (2)
H6A—C6—H6C	109.5	C20—C21—H21	120.6
H6B—C6—H6C	109.5	C22—C21—H21	120.6
O5—C7—C12	115.2 (2)	C23—C22—C21	121.6 (3)
O5—C7—C8	124.7 (2)	C23—C22—N2	119.2 (3)
C12—C7—C8	120.1 (2)	C21—C22—N2	119.1 (3)
C9—C8—C7	119.2 (3)	C24—C23—C22	119.7 (3)
C9—C8—H8	120.4	C24—C23—H23	120.1
C7—C8—H8	120.4	C22—C23—H23	120.1
C10—C9—C8	119.7 (3)	C23—C24—C19	119.2 (3)
C10—C9—H9	120.2	C23—C24—H24	120.4
C8—C9—H9	120.2	C19—C24—H24	120.4
C9—C10—C11	121.7 (3)		
			
C5—O1—C1—O5	−57.9 (3)	C17—O8—C13—O12	−61.2 (3)
C5—O1—C1—C2	60.3 (3)	C17—O8—C13—C14	57.4 (3)
C7—O5—C1—O1	−56.9 (3)	C19—O12—C13—O8	−70.4 (3)
C7—O5—C1—C2	−179.1 (2)	C19—O12—C13—C14	167.2 (2)
O1—C1—C2—O2	65.7 (3)	O8—C13—C14—O9	65.8 (3)
O5—C1—C2—O2	−172.55 (19)	O12—C13—C14—O9	−171.1 (2)
O1—C1—C2—C3	−53.0 (3)	O8—C13—C14—C15	−53.4 (3)
O5—C1—C2—C3	68.7 (2)	O12—C13—C14—C15	69.8 (3)
O2—C2—C3—O3	51.8 (3)	O9—C14—C15—O10	59.1 (3)
C1—C2—C3—O3	170.7 (2)	C13—C14—C15—O10	178.1 (2)
O2—C2—C3—C4	−69.2 (2)	O9—C14—C15—C16	−64.5 (3)
C1—C2—C3—C4	49.7 (3)	C13—C14—C15—C16	54.4 (3)
O3—C3—C4—O4	62.8 (2)	O10—C15—C16—O11	62.7 (3)
C2—C3—C4—O4	−176.03 (18)	C14—C15—C16—O11	−176.24 (19)
O3—C3—C4—C5	−173.46 (19)	O10—C15—C16—C17	−179.1 (2)
C2—C3—C4—C5	−52.3 (3)	C14—C15—C16—C17	−58.1 (3)
C1—O1—C5—C6	177.2 (2)	C13—O8—C17—C18	177.8 (2)
C1—O1—C5—C4	−59.7 (2)	C13—O8—C17—C16	−59.2 (3)
O4—C4—C5—O1	178.34 (19)	O11—C16—C17—O8	178.5 (2)
C3—C4—C5—O1	54.7 (2)	C15—C16—C17—O8	57.9 (3)
O4—C4—C5—C6	−62.5 (3)	O11—C16—C17—C18	−62.4 (3)
C3—C4—C5—C6	173.8 (2)	C15—C16—C17—C18	177.0 (2)
C1—O5—C7—C12	172.6 (2)	C13—O12—C19—C20	−161.3 (2)
C1—O5—C7—C8	−8.9 (4)	C13—O12—C19—C24	19.7 (4)
O5—C7—C8—C9	−179.0 (3)	O12—C19—C20—C21	−179.2 (2)
C12—C7—C8—C9	−0.6 (4)	C24—C19—C20—C21	−0.2 (4)
C7—C8—C9—C10	−1.1 (4)	C19—C20—C21—C22	0.3 (4)
C8—C9—C10—C11	1.7 (5)	C20—C21—C22—C23	−0.2 (4)
C8—C9—C10—N1	−178.5 (3)	C20—C21—C22—N2	179.5 (3)
O6—N1—C10—C9	−13.4 (4)	O14—N2—C22—C23	−0.8 (5)
O7—N1—C10—C9	167.3 (3)	O13—N2—C22—C23	−179.8 (3)
O6—N1—C10—C11	166.4 (3)	O14—N2—C22—C21	179.5 (4)
O7—N1—C10—C11	−13.0 (4)	O13—N2—C22—C21	0.5 (4)
C9—C10—C11—C12	−0.6 (4)	C21—C22—C23—C24	0.0 (5)
N1—C10—C11—C12	179.6 (3)	N2—C22—C23—C24	−179.7 (3)
C10—C11—C12—C7	−1.1 (4)	C22—C23—C24—C19	0.1 (4)
O5—C7—C12—C11	−179.7 (3)	O12—C19—C24—C23	179.0 (3)
C8—C7—C12—C11	1.7 (4)	C20—C19—C24—C23	0.1 (4)

### Hydrogen-bond geometry (Å, °)

**Table:**

*D*—H···*A*	*D*—H	H···*A*	*D*···*A*	*D*—H···*A*
O2—H2A···O15^i^	0.87 (4)	1.83 (4)	2.697 (3)	171 (4)
O3—H3A···O4^ii^	0.87 (4)	1.78 (4)	2.652 (3)	179 (3)
O4—H4A···O10	0.829 (19)	1.98 (2)	2.799 (3)	168 (3)
O9—H9A···O11^iii^	0.80 (4)	1.96 (4)	2.724 (3)	161 (3)
O10—H10···O3^ii^	0.828 (19)	2.18 (2)	2.993 (3)	166 (3)
O11—H11A···O3^ii^	0.816 (19)	1.92 (2)	2.668 (3)	153 (3)
O15—H15A···O9	0.89 (2)	2.04 (2)	2.909 (3)	165 (5)
O15—H15B···O2^ii^	0.88 (2)	1.96 (2)	2.820 (3)	167 (5)

Symmetry codes: (i) *x*−1, *y*, *z*; (ii) −*x*+1, *y*−1/2, −*z*+1; (iii) *x*, *y*+1, *z*.
